# *Actinobacillus pleuropneumoniae* exotoxin ApxI induces cell death via attenuation of FAK through LFA-1

**DOI:** 10.1038/s41598-021-81290-9

**Published:** 2021-01-18

**Authors:** Siou-Cen Li, Yu-Tsen Cheng, Ching-Yang Wang, Jia-Ying Wu, Zeng-Weng Chen, Jyh-Perng Wang, Jiunn-Horng Lin, Shih-Ling Hsuan

**Affiliations:** 1 Graduate Institute of Veterinary Pathobiology, College of Veterinary Medicine, National Chung Hsing University, Taichung City, 402 Taiwan; 2 Animal Technology Laboratories, Agricultural Technology Research Institute, Hsinchu City, 300 Taiwan

**Keywords:** Cell death, Cell signalling, Bacterial pathogenesis, Bacterial toxins

## Abstract

ApxI exotoxin is an important virulence factor derived from *Actinobacillus pleuropneumoniae* that causes pleuropneumonia in swine. Here, we investigate the role of lymphocyte function-associated antigen 1 (LFA-1, CD11a/CD18), a member of the β_2_ integrin family, and the involvement of the integrin signaling molecules focal adhesion kinase (FAK) and Akt in ApxI cytotoxicity. Using Western blot analysis, we found that ApxI downregulated the activity of FAK and Akt in porcine alveolar macrophages (AMs). Preincubation of porcine AMs with an antibody specific for porcine CD18 reduced ApxI-induced cytotoxicity as measured by a lactate dehydrogenase release assay and decreased ApxI-induced FAK and Akt attenuation, as shown by Western blot analysis. Pretreatment with the chemical compounds PMA and SC79, which activate FAK and Akt, respectively, failed to overcome the ApxI-induced attenuation of FAK and Akt and death of porcine AMs. Notably, the transfection experiments revealed that ectopic expression of porcine LFA-1 (pLFA-1) conferred susceptibility to ApxI in ApxI-insensitive cell lines, including human embryonic kidney 293T cells and FAK-deficient mouse embryonic fibroblasts (MEFs). Furthermore, ectopic expression of FAK significantly reduced ApxI cytotoxicity in pLFA-1-cotransfected FAK-deficient MEFs. These findings show for the first time that pLFA-1 renders cells susceptible to ApxI and ApxI-mediated attenuation of FAK activity via CD18, thereby contributing to subsequent cell death.

## Introduction

*Actinobacillus pleuropneumoniae* (App) is a Gram-negative bacterium that causes severe hemorrhagic and necrotizing pleuropneumonia in pigs, leading to great economic loss in industry. App-derived Apx exotoxins, i.e., ApxI-IV, belong to the repeats-in-toxin (RTX) family and are the most important virulence factors involved in the pathogenesis of App^1^. Apx exotoxins exert cytotoxic effects on porcine alveolar macrophages (AMs), neutrophils, and lymphocytes, impairing the host defense mechanisms^2–4^. ApxI has a strong cytotoxic effect and causes cellular damage and apoptosis in porcine AMs, which provide the first line of defense against bacterial infection in the lungs via their phagocytic and lytic abilities^5–8^.

RTX toxins, including the α-hemolysin (Hly) of *Escherichia coli*, leukotoxin (Lkt) of *Mannheimia haemolytica*, leukotoxin (Ltx) of *Aggregatibacter actinomycetemcomitans*, and adenylate cyclase toxin (CyaA) of *Bordetella pertussis*, interact with β_2_ integrin and lead to cell death^9–13^. Human LFA-1 is a receptor for Hly and Ltx, and the binding of either toxin to LFA-1 causes cytolysis^11^. Bovine LFA-1 is a receptor for Lkt, which binds CD11a and CD18 subunits^14^. In addition, β_2_ integrin plays an important role in Apx toxin-induced events. The CD18 subunit of lymphocyte function-associated antigen 1 (LFA-1; CD11a/CD18) results in ApxIII-induced cell death^15^, and CD18 mediates ApxI-induced activation of the p38, c-Jun N-terminal kinase, and nuclear factor-κB pathways for the expression of proinflammatory cytokines^16,17^. However, the current knowledge regarding how β_2_ integrin interacts with Apx toxins and the signaling mechanisms underlying the cytotoxicity of Apx toxins is limited.

β_2_ integrins consist of α (CD11) and β_2_ (CD18) subunits and are expressed primarily on leukocytes to mediate cell adhesion, migration, differentiation, survival, and proliferation^18^. The members of the β_2_ integrin family include LFA-1, macrophage-1 antigen (Mac-1; CD11b/CD18), complement receptor 4 (CD11c/CD18), and αDβ_2_ (CD11d/CD18). Upon ligand binding, inactive β_2_ integrins switch to an active state, interact with adaptor molecules, and initiate a signaling network via α-actinin, 14–3–3 protein, or focal adhesion kinase (FAK), leading to corresponding cellular functions^19^. FAK and Akt are important regulators of integrin-derived survival signals. The role of the FAK signaling pathway in β_1_ integrin-mediated cell survival is well defined, and Akt modulates diverse prosurvival and antideath functions^20^. The activation of prodeath pathways and/or impairment of prosurvival pathways may pave the way for cell death^20,21^.

Although it has been thoroughly demonstrated that activation of the β_1_ integrin-FAK pathway supports cell survival, the role of FAK and Akt in β_2_ integrin-mediated cell survival has not been extensively investigated. Previous studies reported that CyaA, Hly, and Lkt attenuated Akt activity^22–24^, suggesting that RTX toxins downregulate cell survival pathways. Whether LFA-1 mediates the cytotoxic effects of ApxI via FAK and Akt is unclear. Therefore, the present study examined the role of LFA-1 in ApxI cytotoxicity and delineated the possible involvement of FAK and Akt in this event.

## Materials and methods

### Chemicals, reagents, and antibodies

Poly-D-lysine (PDL), polymyxin B, 2,3-bis (2-methoxy-4-nitro-5-sulfophenyl)-2H-tetrazolium-5-carbo-xanilide inner salt (XTT), Hoechst 33342, phorbol 12-myristate 13-acetate (PMA), dimethyl sulfoxide (DMSO), 2-amino-6-chloro-α-cyano-3-(ethoxycarbonyl)-4H-1-benzopyran-4-acetic acid ethyl ester (SC79), bovine serum albumin (BSA), and an anti-β-actin antibody were purchased from Sigma-Aldrich (Merck, Germany). Phosphate-buffered saline (PBS) was purchased from Thermo Fisher Scientific (USA).

The CD11a-specific monoclonal antibodies MUC76A and CD11a-EXT were purchased from Kingfisher Biotech (USA) and AllBio (Taiwan), respectively. The CD18-specific antibodies PNK-1 and LS-C312785 were purchased from Bio-Rad (USA) and Lifespan Biosciences (USA), respectively. Isotype-matched mouse IgG_1_ and IgG_2a_ were purchased from Thermo Fisher Scientific (USA) and Kingfisher Biotech (USA), respectively. The antibodies against phospho-FAK^Tyr397^ and phospho-Akt^Ser473^ were purchased from Cell Signaling Technology (USA), and an antibody recognizing phospho-FAK^Tyr925^ was purchased from Bioss (USA).

### Cell culture

#### Porcine AMs

Porcine AMs were obtained from 6- to 8-week-old specific pathogen-free pigs via lavage and stored in liquid nitrogen using previously described procedures^6^. The protocol for the euthanasia of the pigs was approved by the Institutional Animal Care and Use Committee of Agricultural Technology Research Institute (permit number 10781) and was in accordance with the Guide for the Care and Use of Laboratory Animals (Council of Agriculture, Executive Yuan, ROC) and the ARRIVE Guidelines. Euthanasia was performed by intramuscular injection of azaperone (2 mg/kg body weight; Stroless, China Chemical & Pharmaceutical, Taiwan) and a combination of equal parts by weight of tiletamine and zolazepam (4 mg/kg body weight; Zoletil™ 50, Virbac, France) to induce sedation and anesthesia, respectively, followed by electrocution. Prior to use in experiments, porcine AMs were thawed and incubated at 37 °C in a humidified CO_2_ incubator overnight. The culture medium for porcine AMs was RPMI-1640 supplemented with 10% FBS, 2 mM L-glutamine, 100 U/ml penicillin, and 100 μg/ml streptomycin.

#### Human embryonic kidney (HEK) cells

HEK 293T cells (Clontech, USA) were cultured in Dulbecco's modified Eagle's medium (DMEM) supplemented with 10% FBS, nonessential amino acids, 100 U/ml penicillin, and 100 μg/ml streptomycin at 37 °C in a humidified CO_2_ incubator.

#### Mouse embryonic fibroblasts (MEFs)

FAK-deficient MEFs (ATCC CRL­2644) were cultured in DMEM supplemented with 10% FBS, 100 U/ml penicillin, and 100 μg/ml streptomycin at 37 °C in a humidified CO_2_ incubator.

### Preparation of crude ApxI exotoxin

An isolate of *A. pleuropneumoniae* serotype 10 (strain 13039) was a gift from the Animal Health Research Institute, Council of Agriculture, ROC. The preparation of the exotoxin and measurement of the cytotoxic activity using an XTT assay were performed according to previously described procedures^6^. One cytotoxic unit (CU) of ApxI was defined as the quantity of toxin that caused a 50% reduction in mitochondrial activity in porcine AMs.

### Plasmid preparation

pCX-MCS1 was a gift from Dr. Chin-Kai Chuang (Agricultural Technology Research Institute, Taiwan, ROC). The pCX-CD11a, pCX-CD18 and pCX-GFP plasmids were constructed by separately cloning the coding sequence of porcine CD11a (GenBank accession DQ013284) with *Xho*I and *Hin*dIII linkers, the coding sequence of porcine CD18 (GenBank accession U13941) with *Xho*I and *Kpn*I linkers, and the coding sequence of green fluorescent protein (National Center for Biotechnology Information reference sequence WP_153939948) with *Eco*RI and *Hin*dIII linkers into the multiple cloning site in pCX-MCS1. The FAK expression plasmid and corresponding control plasmid were purchased from OriGene (USA). The plasmids were transformed into *E. coli* TOP10 (Invitrogen, USA) and prepared from bacterial cultures using a Hispeed Plasmid Midi Kit (Qiagen, Germany) according to the manufacturer’s instructions.

### Transfection

HEK 293T cells or MEFs were seeded in PDL-coated 35-mm cell culture plates (1 × 10^6^ HEK 293T cells or 2 × 10^5^ MEFs/plate) or PDL-coated 12-mm coverslips in a 24-well plate (1 × 10^5^ HEK 293T cells/well) and incubated at 37 °C in a 5% CO_2_ atmosphere overnight. The culture medium was replaced with medium without antibiotics on the day of transfection. Lipofectamine 2000 (Invitrogen, USA) was used for transfection according to the manufacturer’s instructions. For confocal microscopy, HEK 293T cells on coverslips were transfected with 0.5 μg of pCX-CD11a and 0.5 μg of pCX-CD18, 1 μg of pCX-CD11a, 1 μg of pCX-CD18, or 1 μg pCX-MCS1. For transfection of HEK 293T cells for the LDH release assay or Western blot analysis, HEK 293T cells in 35-mm plates were transfected with 2 μg of pCX-CD11a and 2 μg of pCX-CD18 or 4 μg of pCX-GFP. For MEF transfection, the cells were transfected with 1.5 μg of pCX-CD11a and 1.5 μg of pCX-CD18 along with 0.3 μg of the FAK-expressing plasmid or corresponding control plasmid or with 3.3 μg of the corresponding empty vector as the control group. HEK 293T cells or MEFs were transfected for 24 h prior to subsequent experiments.

### Treatment with ApxI, drugs, and antibodies

For experiments with ApxI treatment, cells were washed with low-serum medium (LSM; RPMI-1640 supplemented with 2 mM L-glutamine, 1% FBS, 100 IU/ml penicillin, 100 µg/ml streptomycin, and 10 µg/ml polymyxin B), and incubated with with ApxI in LSM. Transfected HEK 293T cells and MEFs were incubated with 0–25 CU/ml and 0–8 CU/ml ApxI, respectively, for 5 h for LDH release assay. Porcine AMs without activator or antibody pretreatment were incubated with 0 or 2.5 CU/ml ApxI for 0–60 min for Western blot analysis. In experiments with activator treatment, porcine AMs were incubated with LSM containing 200 nM PMA, 4 µg/ml SC79, or 0.1% DMSO for 0, 15, 30, or 60 min prior to Western blot analysis. In experiments with activator pretreatment and ApxI treatment, porcine AMs were incubated with LSM containing 200 nM PMA, 4 µg/ml SC79, or 0.1% DMSO for 15 min prior to incubation with ApxI. For Western blot analysis, PMA- and SC79-pretreated porcine AMs were incubated with 0 or 2.5 CU/ml ApxI for 0–5 min and 0–60 min, respectively. For LDH release assay, PMA- and SC79-treated porcine AMs were incubated for 90 min with 0–10 CU/ml and 0–5 CU/ml ApxI, respectively. In experiments with antibody pretreatment, porcine AMs were incubated with LSM containing 10 μg/ml of a monoclonal antibody (MUC76A, PNK-1, or isotype control) on ice for 60 min prior to incubation with 2 CU/ml ApxI for 8 h, and 0 or 2.5 CU/ml ApxI for 10 min for LDH release assay and Western blot analysis, respectively. To minimize LPS contamination in the exotoxin preparation, polymyxin B was added to a final concentration of 10 μg/ml throughout this study.

### Lactate dehydrogenase (LDH) release assay

ApxI-induced cell death was measured as the activity of LDH released from damaged cells using a cytotoxicity detection kit (Roche, Switzerland). Briefly, porcine AMs or transfected cells were detached using Accutase (Gibco), suspended in LSM, and seeded in 96-well culture plates at a density indicated elsewhere. The cells were treated with ApxI for the indicated periods, and the cell culture supernatant was collected by centrifugation at 200 × *g* for 10 min. The level of LDH activity in the cultural supernatant and the cytotoxicity percentage were determined according to the manufacturer’s instructions.

### Confocal microscopy

Transfected HEK 293T cells on PDL-coated coverslips were washed with PBS, fixed with 1% paraformaldehyde for 10 min, incubated with 2 mg/ml ammonium chloride in PBS containing 0.05% Tween-20 for 10 min, and blocked with 5% BSA in PBS for 30 min. The cells were incubated with monoclonal antibodies MUC76A and PNK-1 (1 μg/ml of each antibody) for 30 min at 4 °C, followed by incubation with Alexa Fluor 488- and PE-conjugated secondary antibodies. The nuclei were stained with 1 µg/ml Hoechst 33342 for 15 min. The cell surface expression of LFA-1 was evaluated under a confocal microscope (FV1000; Olympus, Japan).

### Western blot analysis

Cell lysates were collected via centrifugation at 14,000 × *g*, and the protein concentrations were determined according to a previously described procedure^16^. SDS–polyacrylamide gel electrophoresis was used to separate 20–50 μg of cell lysate, and the separated proteins were transferred to polyvinylidene difluoride membranes. The membranes were probed with primary antibodies specific for porcine CD11a, porcine CD18, phospho-FAK^Tyr397^, phospho-FAK^Tyr925^, phospho-Akt^Ser473^, or β-actin and were then incubated with the corresponding secondary antibody. The intensities of the immunoreactive bands were quantified using ImageJ software (National Institutes of Health, USA) and normalized to the intensity of the loading control β-actin.

### Statistical analysis

Data obtained from at least three independent experiments were analyzed using Student’s *t*-test. GraphPad Prism 6 (GraphPad) was used for statistical analysis. The error bars indicate the standard deviations. A difference was considered significant when *p* < 0.05.

## Results

### Ectopically expressed porcine LFA-1 confers susceptibility to ApxI in human embryonic kidney 293T cells

**Figure 1:**
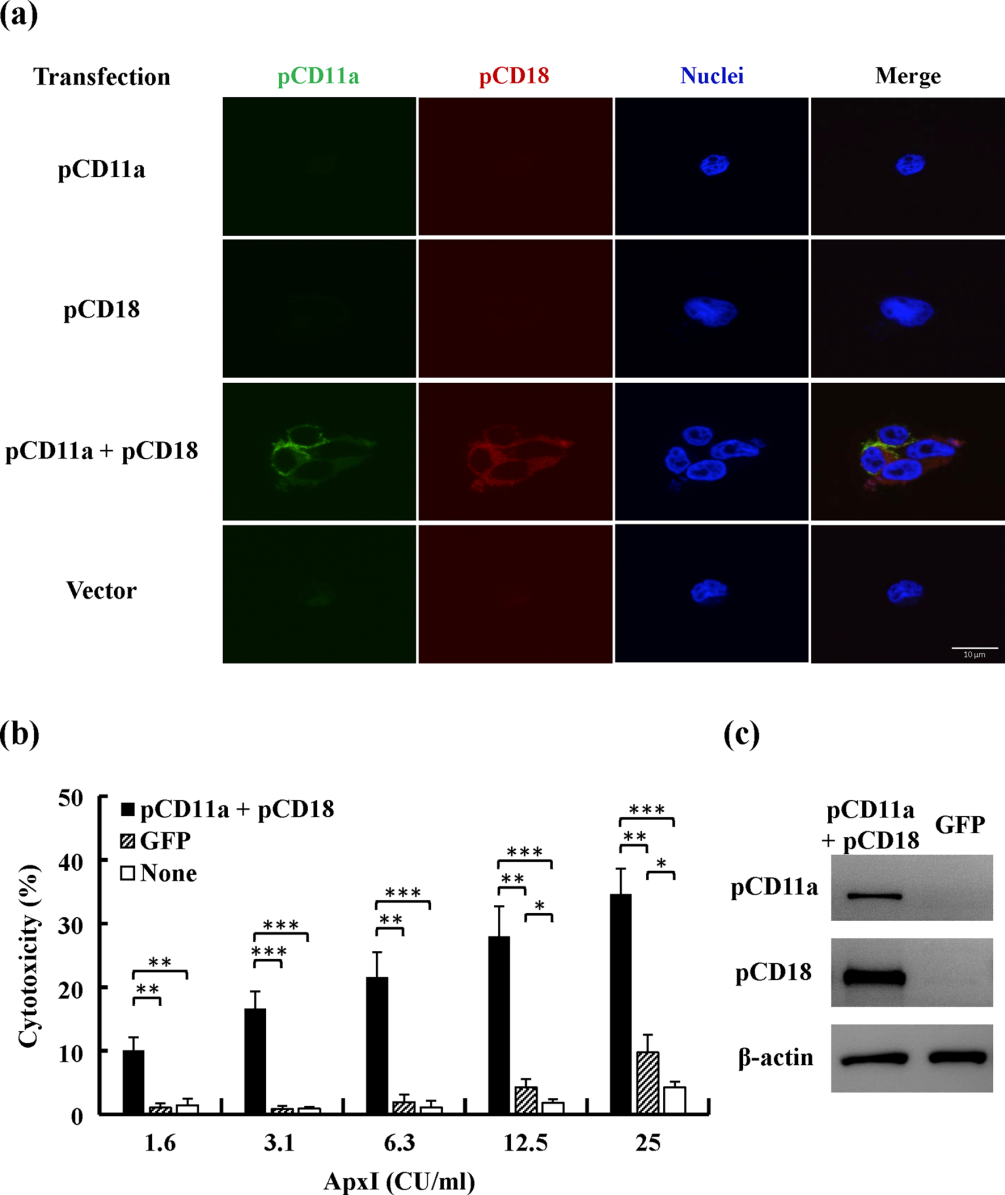
Porcine LFA-1 confers susceptibility to ApxI in HEK 293T cells. (**a**) HEK 293T cells were transfected with plasmids expressing porcine CD11a (pCD11a) and/or porcine CD18 (pCD18) or the corresponding empty vector. Ectopic expression of pCD11a and pCD18 was examined using confocal microscopy. Confocal images show the subcellular localization of pCD11a (pseudogreen), pCD18 (pseudored), and nuclei (pseudoblue). (**b**) Cells were transfected with plasmids expressing pCD11a and pCD18, green fluorescent protein (GFP), or non-transfected (None) for 24 h, followed by incubation with 1.6–25 CU/ml of ApxI for 5 h. The culture supernatants were collected and analyzed using the LDH release assay. Data are from three independent experiments of triplicate determinants. Error bars represent the standard deviations. *p < 0.05; **p < 0.01; ***p < 0.001. (**c**) Cells were transfected with plasmids expressing pCD11a and pCD18 or GFP. Expression of pCD11a and pCD18 was examined using Western blot analysis. Immunoblots were also probed with an anti-β-actin antibody as the loading control. The grouping of blots was cropped from different portions of the same gel and exposed separately. Uncropped blot images for (**c**) are presented in Supplementary Figure S1.

To examine the role of porcine LFA-1 (pLFA-1, pCD11a/pCD18) in ApxI cytotoxicity, ApxI-insensitive HEK 293T cells were used. First, the HEK 293T cells were transfected with a plasmid carrying porcine *cd11a*, *cd18* or cotransfected with both plasmids for 24 h and subjected to confocal microscopy analyses for the expression of pCD11a and pCD18. pCD11a and pCD18 were simultaneously detected primarily at the cell membrane in pCD11a/pCD18 cotransfected cells. In contrast, neither pCD11a nor pCD18 was detected at the cell membrane in HEK 293T cells transfected only with a plasmid encoding pCD11a or pCD18 (Fig. 1a). Subsequently, cytotoxicity of ApxI was investigated in HEK 293T cells cotransfected with plasmids expressing pCD11a and pCD18, green fluorescent protein (GFP), and non-transfected cells. These cells were treated with 1.6 to 25 CU/ml of ApxI for 5 h. The level of LDH released to the culture medium was measured as an indicator of cell membrane damage and/or cytolysis. ApxI-induced cytotoxicity in the pCD11a/pCD18-cotransfected cells was significantly higher than the control groups, including the GFP-transfected cells and the nontransfected cells. The levels of cytotoxicity were 10–35% at 1.6–25 CU/ml ApxI in the pCD11a/pCD18-cotransfected cells, and 1–10% in the controls (Fig. 1b). Cotransfection of pCD11a/pCD18 led to a 3.5- to 11-fold increase in cytotoxicity levels to ApxI compared to control groups. The expression of pCD11a and pCD18 in cotransfected HEK 293T cells was confirmed using Western blot analysis (Fig. 1c). Taken together, cotransfection of porcine *cd11a* and *cd18* was essential for pLFA-1 expression on the cell surface of HEK 293T cells. pLFA-1 is pivotal to ApxI cytotoxicity, as evidenced by the findings that ectopic expression of pLFA-1 rendered ApxI-insensitive cells susceptible to ApxI.

### CD18 mediates the cytotoxicity of ApxI in porcine AMs

**Figure 2:**
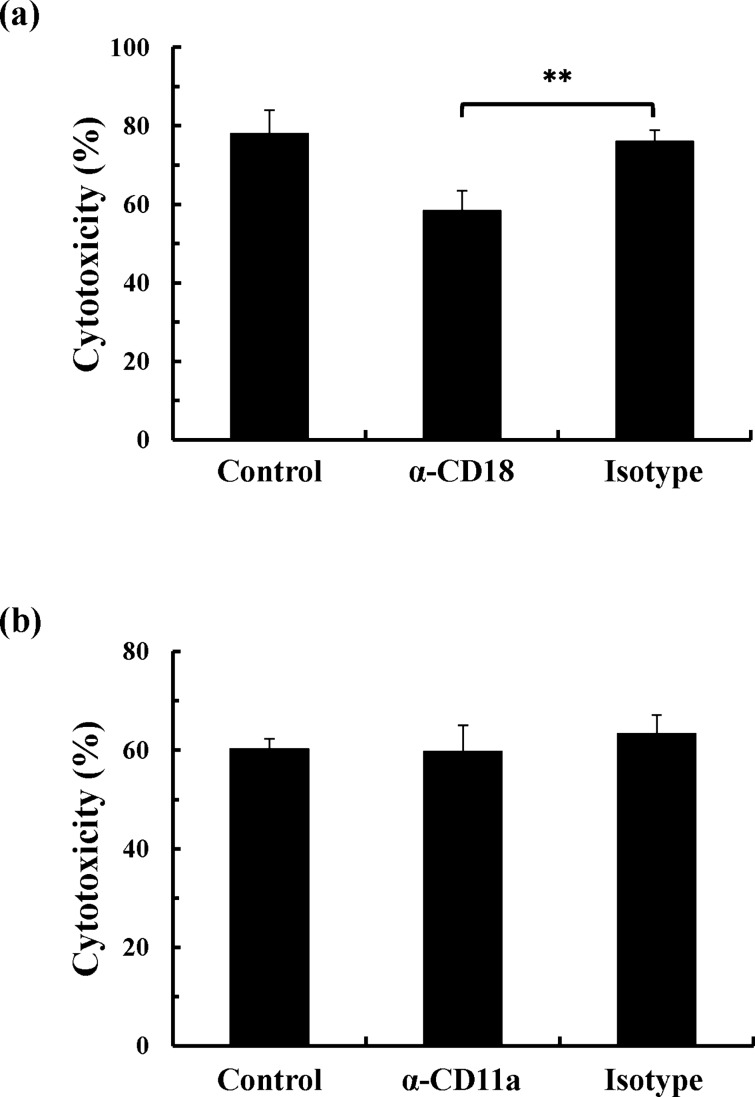
CD18 is involved in ApxI-induced cytotoxicity in porcine AMs. Porcine AMs were preincubated with medium (Control), (**a**) anti-CD18 antibody (α-CD18), (**b**) anti-CD11a antibody (α-CD11a), or isotype-matched control (Isotype) on ice for 1 h. The cells were incubated with 2 CU/ml of ApxI for an additional 8 h, and the culture supernatants were subjected to LDH release assay. The error bars represent the standard deviations. **p < 0.01. Data are from three independent experiments of triplicate determinants.

To evaluate the involvement of the two subunits of LFA-1 in ApxI-induced cell death, porcine AMs were preincubated with specific antibodies against CD11a or CD18 for 1 h and treated with 2 CU/ml of ApxI for 8 h, and the level of LDH release was measured. Preincubation with a CD18 antibody significantly reduced ApxI-induced cytotoxicity by 18% (Fig. 2a). However, ApxI-induced cytotoxicity in cells preincubated with a CD11a antibody was not affected (Fig. 2b). These findings suggest that CD18 serves as an important mediator of ApxI-induced cell death in porcine AMs.

### ApxI attenuates FAK and Akt activity in porcine AMs

**Figure 3:**
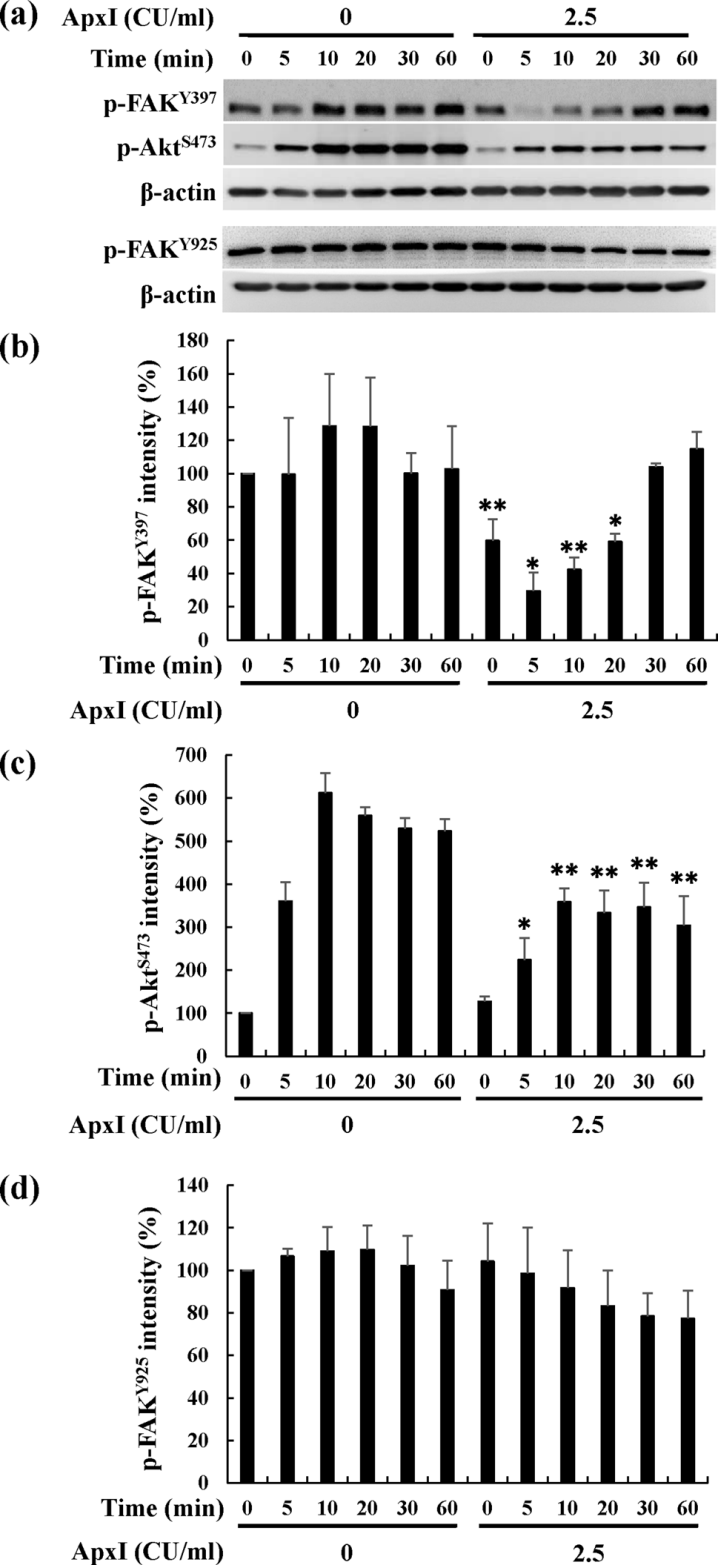
ApxI decreases FAK and Akt activity in porcine AMs. Porcine AMs were treated with 0 or 2.5 CU/ml ApxI for 0–60 min. Cell lysates were collected and subjected to (**a**) Western blot analyses for phospho-FAK^Tyr397^ (p-FAK^Tyr397^), phospho-FAK^Tyr925^ (p-FAK^Y925^), phospho-Akt^Ser473^ (p-Akt^S473^), and β-actin. The grouping of blots was cropped from different portions of two gels and exposed separately. Uncropped blot images for Fig. 3(**a**) are presented in Supplementary Figure S2. The average intensities of (**b**) p-FAK^Tyr397^, (**c**) p-Akt^Ser473^, and (**d**) p-FAK^Tyr925^ were from three independent experiments and normalized to the intensity of β-actin. Asterisks indicate significant differences compared to the non-ApxI treatment group at the identical time point. *p < 0.05; **p < 0.01.

A plethora of studies indicate that the integrin signaling molecules, such as FAK, phosphoinositide 3-kinase (PI3K) and Akt, play important roles in cell survival. To investigate whether FAK was involved in ApxI cytotoxicity, FAK activity in porcine AMs treated with ApxI for 0–60 min was examined using Western blot analysis to assess the levels of phospho-FAK^Tyr397^ (p-FAK^Tyr397^) and phospho-FAK^Tyr925^ (p-FAK^Tyr925^). Upon exposure to 2.5 CU/ml of ApxI, the level of p-FAK^Tyr397^ decreased significantly during the first 20 min, but it was unaffected at the same time points in cells treated with control medium (0 CU/ml of ApxI) (Fig. 3a,b). The level of p-FAK^Tyr397^ declined significantly to 29% at 5 min of exposure to ApxI, then recovered to the basal level at 30 min. The level of p-FAK^Tyr925^ decreased to 77% during 1 h-exposure to ApxI, which did not differ significantly from the time-matched control (Fig. 3a,d). Akt activity in ApxI-treated porcine AMs was also examined using detection of the phospho-Akt^Ser473^ (p-Akt^Ser473^) level. The analytical results showed that the level of p-Akt^Ser473^ increased after 5 min of incubation regardless of the presence or absence of ApxI treatment (Fig. 3a,c). The level of p-Akt^Ser473^ in ApxI-treated cells increased 3-fold after a 10-min ApxI incubation and remained constant for 60 min, and these levels were significantly lower than the control medium-treated group. Taken together, the time course studies reveal that ApxI significantly attenuated the activity of FAK and Akt in porcine AMs.

### ApxI-mediated attenuation of FAK/Akt activity requires CD18 in porcine AMs

**Figure 4:**
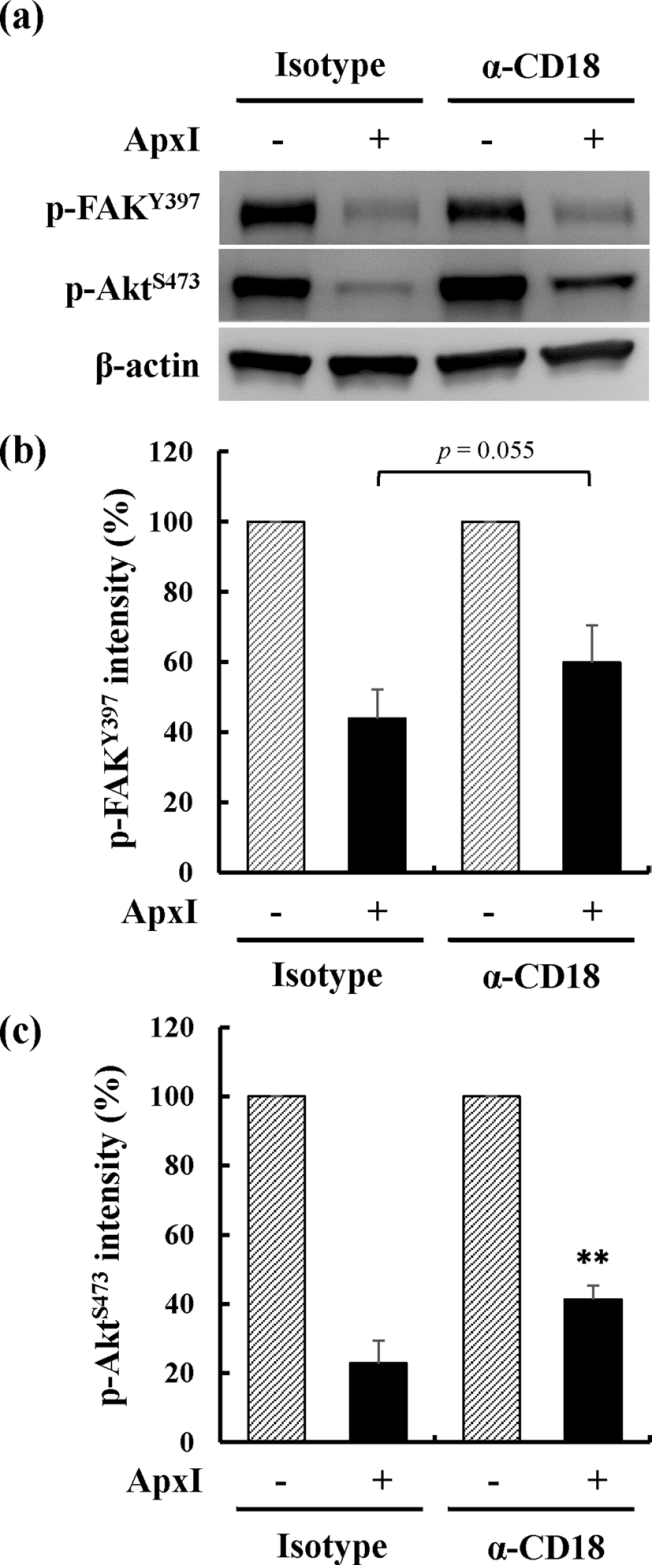
Pretreatment with a CD18-specific antibody reduces ApxI-induced decreases in the levels of p-FAK^Tyr397^ and p-Akt^Ser473^ in porcine AMs. Porcine AMs were preincubated with an anti-CD18 antibody (α-CD18) or isotype control (Isotype) on ice for 1 h. ApxI was added to a final concentration of 2.5 CU/ml for an additional 10 min, and cell lysates were subjected to (**a**) Western blot analysis for p-FAK^Y397^, p-Akt^S473^, and β-actin. The grouping of blots was cropped from different portions of the same gel and exposed separately. Uncropped blot images for (**a**) are presented in Supplementary Figure S3. The average intensities of (**b**) p-FAK^Tyr397^ and (**c**) p-Akt^Ser473^ are from three independent experiments and normalized to the intensity of β-actin. The intensity of the ApxI-treated group was further normalized to the intensity of the non-ApxI-treated group. Asterisks indicate significant differences compared to the isotype control. **p < 0.01.

To examine whether CD18 was involved in ApxI-mediated attenuation of FAK/Akt activity, porcine AMs were preincubated with a CD18-specific antibody for 1 h and treated with 2.5 CU/ml ApxI for 10 min. The levels of p-FAK^Tyr397^ and p-Akt^Ser473^ were examined using Western blot analysis. The analytical results showed that the levels of p-FAK^Tyr397^ were 44% and 60% in the ApxI-treated cells with prior incubation with an isotype-matched antibody and CD18 antibody, respectively (Fig. 4a,b). Similarly, the levels of p-Akt^Ser473^ in ApxI-treated cells were 41% with CD18 antibody preincubation compared to the isotype control, which was 23% (Fig. 4a,c). Collectively, a CD18-specific antibody reversed the ApxI-induced reduction in FAK and Akt activity, which suggests a role of CD18 in mediating ApxI-induced FAK/Akt attenuation in porcine AMs.

### PMA and SC79 fail to restore the activity of FAK and Akt, respectively, and rescue ApxI-induced cell death in porcine AMs

We hypothesized that if the reductions of FAK and Akt activity were keys to ApxI-induced cell death, then enhancement of FAK or Akt activity would protect cells from ApxI-induced cell death. Therefore, we examined whether ApxI-induced FAK and Akt attenuation and cell death were modulated via chemical activators of FAK or Akt, i.e., PMA and SC79, respectively.

**Figure 5:**
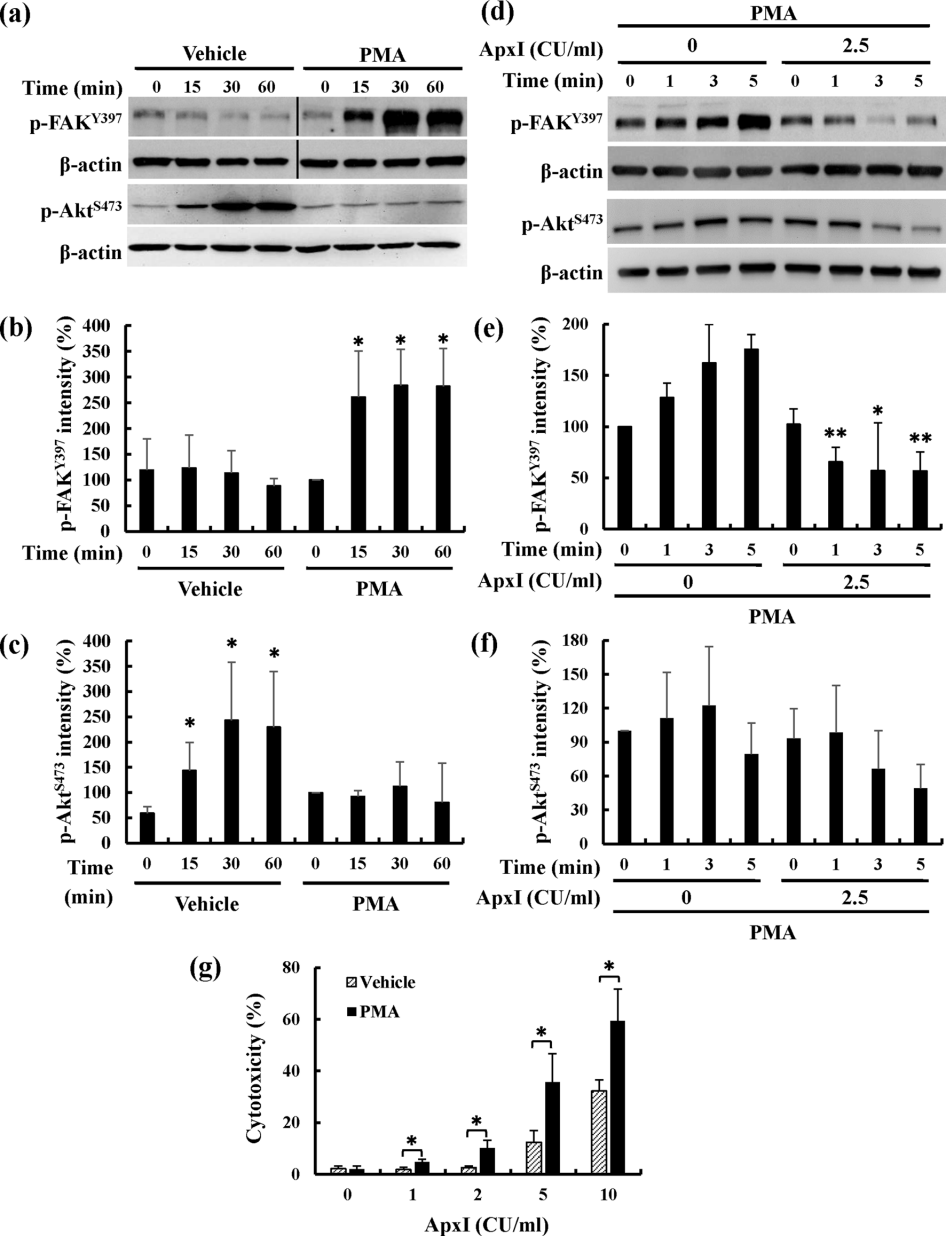
PMA fails to overcome ApxI-induced reduction in FAK and Akt activity and cytotoxicity. (**a**–**c**) Porcine AMs were treated with 200 nM PMA or 0.1% DMSO (Vehicle) for 0–60 min. Cell lysates were subjected to (**a**) Western blot analyses for p-FAK^Y397^ and p-Akt^S473^. The grouping of blots was cropped from different portions of two gels and exposed separately. Black lines delineate the boundary between not contiguous lanes of the same gel. Uncropped blot images for (**a**) are presented in Supplementary Figure S4. The average intensities of (**b**) p-FAK^Tyr397^ and (**c**) p-Akt^Ser473^ are from three independent experiments and normalized to the intensity of β-actin. Asterisks indicate significant differences compared to the 0-min treatment group. *p < 0.05. (**d–f**) Porcine AMs pretreated with 200 nM PMA for 15 min were stimulated with 0 or 2.5 CU/ml of ApxI for 0–5 min. Cell lysates were collected and subjected to (**d**) Western blot analyses for p-FAK^Y397^, p-Akt^S473^, and β-actin. The grouping of blots was cropped from different portions of two gels and exposed separately. Uncropped blot images for (**d**) are presented in Supplementary Figure S5. The average intensities of (**e**) p-FAK^Tyr397^ and (**f**) p-Akt^Ser473^ are from three independent experiments and normalized to the intensity of β-actin. Asterisks indicate significant differences compared to the non-ApxI treatment group at the identical time point. *p < 0.05; **p < 0.01. (**g**) Porcine AMs were incubated with 200 nM PMA or 0.1% DMSO (Vehicle) for 15 min, then incubated with 0–10 CU/ml of ApxI for 90 min. The percent cytotoxicity was quantified using the LDH release assay. Data are from three independent experiments of triplicate determinants. *p < 0.05.

To test the effect of PMA on FAK activation, porcine AMs were incubated with PMA for 0–60 min, and the activity of FAK and Akt was determined using Western blot analysis. The levels of p-FAK^Tyr397^ increased significantly 2.6-fold after a 15-min PMA treatment and remained constant for up to 60 min of PMA treatment (Fig. 5a,b). However, the level of p-Akt^Ser473^ decreased after PMA treatment (Fig. 5a,c). These results indicated that PMA activated FAK, but not Akt, activity in porcine AMs. To investigate the effects of PMA on ApxI-mediated attenuation of FAK and Akt activity, porcine AMs were incubated with PMA prior to ApxI stimulation, and the activity of FAK and Akt was determined. A steady increase in p-FAK^Tyr397^ was detected in ApxI-untreated cells pretreated with PMA (Fig. 5d,e). However, the level of p-FAK^Tyr397^ in ApxI-treated cells was reduced 56% after 5 min of exposure to ApxI. The levels of p-Akt^Ser473^ in ApxI-treated groups were 7–56% lower than the time matched, ApxI-untreated groups, but the differences between the ApxI-treated and -untreated groups were statistically insignificant. (Fig. 5d,f). To further examine the effect of PMA on ApxI cytotoxicity, porcine AMs preincubated with PMA or DMSO (vehicle) were stimulated with ApxI, and cell death was evaluated. After exposure to 1–10 CU/ml ApxI, the cytotoxicity levels were 2–32% and 5–59% in vehicle- and PMA-treated groups, respectively (Fig. 5g). PMA pretreatment produced a 1.5- to 2.3-fold increase in ApxI cytotoxicity compared to the vehicle pretreatment group. Taken together, PMA did not reverse ApxI-induced attenuation of the levels of p-FAK^Tyr397^ and p-Akt^Ser473^ and failed to rescue cells from ApxI cytotoxicity.

**Figure 6:**
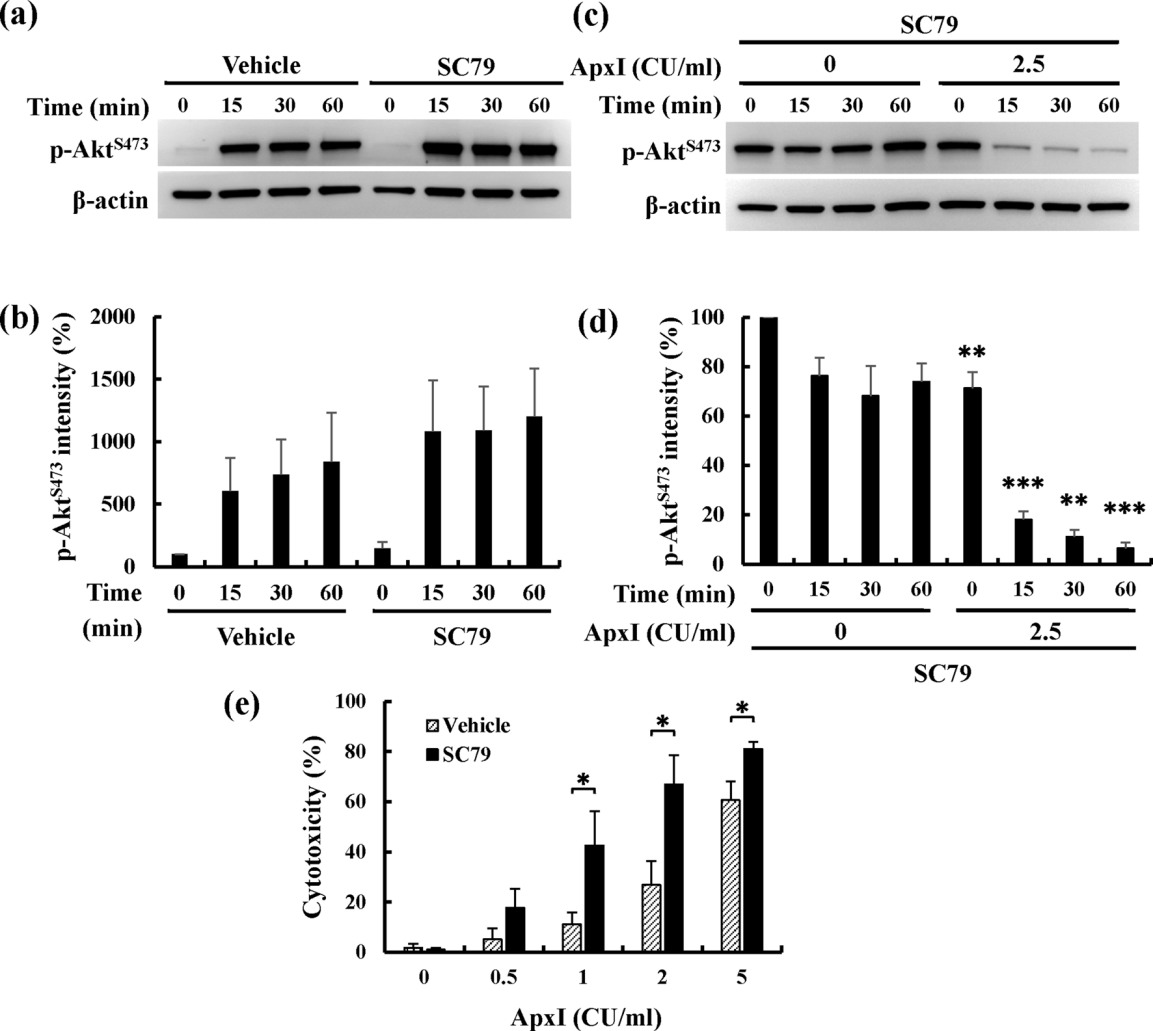
SC79 fails to overcome ApxI-induced attenuation of Akt and cytotoxicity. (**a**,**b**) Porcine AMs were treated with 4 μg/ml SC79 or 0.1% DMSO (Vehicle) for 0–60 min. Cell lysates were subjected to (**a**) Western blot analyses for p-Akt^S473^. The grouping of blots was cropped from different portions of the same gel and exposed separately. Uncropped blot images for (**a**) are presented in Supplementary Figure S6. The average intensity of (**b**) p-Akt^Ser473^ is from three independent experiments and normalized to the intensity of β-actin. (**c**,**d**) Porcine AMs pretreated with 4 μg/ml SC79 for 15 min were incubated with 0 or 2.5 CU/ml of ApxI for 0–60 min. Cell lysates were harvested and subjected to (**c**) Western blot analyses for p-Akt^S473^. The grouping of blots was cropped from different portions of the same gel and exposed separately. Uncropped blot images for (**c**) are presented in Supplementary Figure S7. The average intensity of (**d**) p-Akt^Ser473^ is from three independent experiments and normalized to the intensity of β-actin. Asterisks indicate significant differences compared to the non-ApxI treatment group at the identical time point. (**e**) Porcine AMs were incubated with 4 μg/ml SC79 or 0.1% DMSO (Vehicle) for 15 min, then stimulated with 0–5 CU/ml of ApxI for 90 min. The percent cytotoxicity was quantified using the LDH release assay. Data are from three independent experiments of triplicate determinants. *p < 0.05; **p < 0.01; ***p < 0.001.

To test the effect of SC79 on Akt activity, porcine AMs were incubated with SC79 for 0–60 min, and the activity of Akt was determined using Western blot analysis. The levels of p-Akt^Ser473^ increased 10- to 12-fold after SC79 treatment and increased 6- to 8-fold in the vehicle control group (Fig. 6a,b). SC79 treatment increased Akt activity 1.5-fold compared to vehicle control. To further investigate the effect of SC79 on ApxI-induced attenuation of Akt activity, porcine AMs pretreated with SC79 were stimulated with ApxI, and the level of p-Akt^Ser473^ was examined. The results showed a significant decrease in the level of p-Akt^Ser473^ within 1 h of exposure to ApxI (Fig. 6c,d). These levels fell to 18.1% at 15 min and declined to 6.5% at 1 h. The level of p-Akt^Ser473^ was approximately 73% in the time-matched controls. SC79 did not overcome the ApxI-induced decrease in the level of p-Akt^Ser473^. To further examine the effect of SC79 on ApxI-induced cell death, porcine AMs pretreated with SC79 were stimulated with 0–5 CU/ml ApxI for 90 min, followed by the LDH release assay. The levels of cytotoxicity increased significantly 20–40% in porcine AMs pretreated with SC79 compared to porcine AMs pretreated with vehicle (Fig. 6e). SC79 also failed to reduce ApxI-induced cytotoxicity in porcine AMs.

Collectively, PMA and SC79 failed to restore the FAK and Akt activity affected by ApxI, respectively, and rescue cell death from ApxI treatment.

### Ectopic expression of FAK decreases ApxI-induced effects

**Figure 7:**
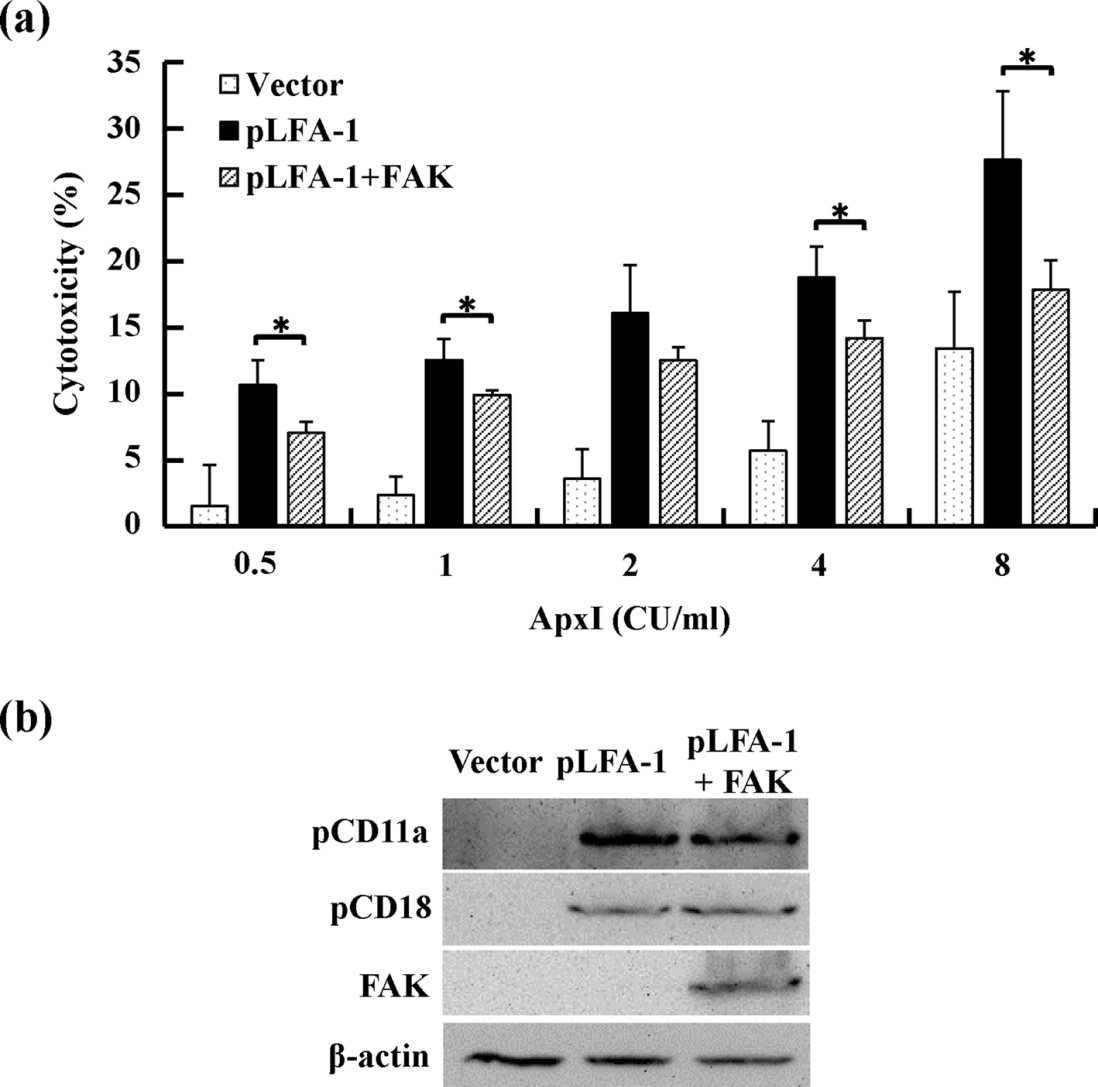
Ectopic expression of FAK attenuates ApxI-induced cytotoxicity in pLFA-1-transfected FAK-deficient MEFs. (**a**) FAK-deficient MEFs were transfected with plasmids expressing porcine LFA-1 (pLFA-1), in combination with a plasmid encoding FAK (pLFA-1 + FAK) or the corresponding empty vectors (Vector) for 24 h. The transfected cells were incubated with ApxI for 5 h. The culture supernatants were collected and analyzed using the LDH release assay. Data are from three independent experiments of triplicate determinants. *p < 0.05. (**b**) FAK-deficient MEFs were transfected with plasmids expressing porcine LFA-1 (pLFA-1) alone, in combination with a plasmid encoding FAK (pLFA-1 + FAK), or the corresponding empty vectors (Vector). Levels of pCD11a, pCD18, and p-FAK^Y397^ were examined using Western blot analyses. Immunoblots were also probed with an anti-β-actin antibody as the loading control. The grouping of blots was cropped from different portions of the same gel and exposed separately. Uncropped blot images for (**b**) are presented in Supplementary Figure S8.

To test whether enhancement of FAK activity via ectopic expression protected cells from ApxI cytotoxicity, FAK-deficient MEFs were transfected with multiple plasmids expressing FAK, pCD11a, and pCD18, or the corresponding empty vectors. The transfected cells were stimulated with 0–8 CU/ml of ApxI for 5 h, and the levels of LDH release were measured. The analytical results showed that the levels of cytotoxicity were 11–28% at 0.5–8 CU/ml ApxI in the pLFA-1-transfected cells and 2–13% in the vector controls. A 2- to 7-fold increase in cytotoxicity levels was observed in the pLFA-1-transfected cells compared to vector controls (Fig. 7a). The levels of cytotoxicity in the cells cotransfected with pLFA-1 and FAK were 7–18% at 0.5–8 CU/ml ApxI, which was significantly lower than the pLFA-1-transfected cells (Fig. 7a). The ectopic expression of pLFA-1 and FAK were confirmed in FAK-deficient MEFs transfected with pCD11a and pCD18 with or without FAK (Fig. 7b). Taken together, the results indicated that pLFA-1 conferred sensitivity to ApxI, and ectopic FAK ameliorated ApxI cytotoxicity in FAK-deficient MEFs.

## Discussion

Integrin signaling plays a critical role in cell survival, cell proliferation, cell motility, cytoskeletal organization, cell differentiation and gene expression^25^. FAK is a central coordinator of integrin signaling and interacts with various signaling molecules to regulate multiple biological processes. The prosurvival role of β_1_ integrin/FAK signaling has been extensively investigated and found to occur via the activation of downstream Src, PI3K and Akt to suppress apoptosis in anchorage-dependent cells^20^. β_2_ integrin Mac-1–derived signals suppress neutrophil apoptosis via the Akt and ERK survival pathway^26^ and regulate the survival and proliferation of human acute myeloid leukemia cells via Syk/STAT activation^27^. To the best of our knowledge, the present study is the first to show that ApxI induces cytotoxicity via the β_2_ integrin LFA-1 and attenuation of the FAK survival signaling protein.

LFA-1 mediates ApxI-induced cell death, as our study showed that cotransfection of porcine *cd11a* and *cd18* conferred susceptibility to ApxI in HEK 293T cells and MEFs. Our finding that singly expressed pCD11a or pCD18 was undetectable on the cell surface further suggests that the coexistence of the pCD11a and pCD18 subunits is essential for the proper dimerization and localization of LFA-1 at the cell membrane. A similar finding of the interdependence of CD11a and CD18 for cell surface expression was reported in human T cells^28^. Because cell surface-expressed LFA-1 mediates ApxI cytotoxicity, LFA-1 likely functions as a receptor for ApxI. LFA-1 is a receptor for other RTX toxin family members, including *E. coli* Hly, *A. actinomycetemcomitans* Ltx, and *M. haemolytica* Lkt^11,14^. Further investigation is required to examine the interaction between ApxI and LFA-1 in order to demonstrate the ligand/receptor interaction. Our current and past studies consistently showed that the CD18 subunit mediated the ApxI-induced effects, including cell death and the secretion of proinflammatory cytokines, in porcine AMs^16,17^. Vanden Bergh et al. also showed that the CD18 subunit of LFA-1 played a pivotal role in ApxIII-induced leukocytolysis^15^. Because CD18 is a common subunit among β_2_ integrin family members, the possible involvement of other β_2_ integrin members in the effects of Apx cannot be excluded.

The present study identified the ApxI-induced attenuation of FAK and Akt in porcine AMs. The levels of p-FAK^Tyr397^ and p-Akt^Ser473^ were significantly reduced shortly after exposure to ApxI. Phosphorylation at Tyr397 is an important step in FAK activation and its subsequent interaction with Src and other downstream signaling molecules to transduce the integrin signal supporting cell survival^20,29^. Decreases in FAK and Akt activity likely indicate the first wave of ApxI-induced signaling events. Comparison of the kinetics of FAK and Akt attenuation upon ApxI exposure suggested that FAK was downstream of β_2_ integrin and upstream of Akt in ApxI-modulated signaling. However, ApxI may attenuate the activity of FAK and Akt via independent pathways, a possibility that warrants further study.

Based on our finding that ApxI attenuated FAK/Akt activity, we hypothesized that downregulation of FAK/Akt signaling could lead to cell death and that an increase in FAK/Akt activity could rescue cells from ApxI cytotoxicity. We used the chemical activators PMA and SC79 to increase FAK and Akt activity, respectively. However, these activators failed to overcome the ApxI-induced attenuation of FAK and Akt activity and enhanced the consequent cell death. PMA significantly increased FAK activation in our study but had an inhibitory effect on the basal activity of Akt. The effect of PMA on Akt activity was inconsistent in various cell types and involved different regulatory mechanisms. For example, PMA increases the level of p-Akt^Ser473^ in breast cancer cells and mast cells via PKCδ activation and interaction with PKCβ, respectively^30,31^, but PMA attenuates Akt activity in endometrial cancer cells and mouse keratinocytes via PKCα, and PKCδ and PKCϵ^32,33^. The present study suggests that the inhibitory effect of PMA on the basal level of Akt activity may explain the potentiation of cell death after ApxI stimulation.

Downregulation of Akt by RTX toxins has been reported previously. *M. haemolytica* Lkt toxin attenuated the level of p-Akt^Ser473^, and *B. pertussis* CyaA and *E*. *coli* Hly reduced the level of p-Akt^Thr308^ and p-Akt^Ser473^^22–24^. Consistent with these studies, we also identified ApxI-induced attenuation of Akt. We further evaluated the ability of the Akt activator SC79 to reverse ApxI cytotoxicity. In contrast to our prediction, SC79 failed to increase Akt activity in response to ApxI and enhanced ApxI-induced cell death. The enhancement of ApxI-induced cell death by SC79 may correspond to the extremely low level of Akt activity. Wiles et al. also reported that the inhibitory effect of *E. coli* Hly on Akt was not overcome by administration of tumor necrosis factor-α and epidermal growth factor or by overexpression of constitutively active Akt mutants, which enabled PtdIns(3,4,5)P_3_-independent translocation or activation of Akt^23^. However, inhibitors of protein phosphatases PP1 and PP2A reduced the attenuation of p-Akt^Ser473^ by Hly. Their study revealed that Hly attenuated Akt activity via the participation of PP1 and PP2A. Whether similar mechanisms are active during ApxI-induced Akt attenuation is unclear; however, our data consistently suggest that ApxI downregulates the activity of FAK and Akt prior to cell death regardless of the presence or absence of PMA or SC79, respectively.

The present study used an alternative approach to determine the role of FAK in ApxI cytotoxicity and evaluate the effect of ectopically expressed FAK in FAK-deficient MEFs. We found that FAK acted as a prosurvival signaling molecule, because the expression of ectopic FAK decreased ApxI-induced cell death in FAK-deficient MEFs transfected with pLFA-1. Notably, pLFA-1-transfected FAK-deficient MEFs remained susceptible to ApxI, suggesting that other unidentified mechanisms may underlie the cytotoxic effect of ApxI.

**Figure 8:**
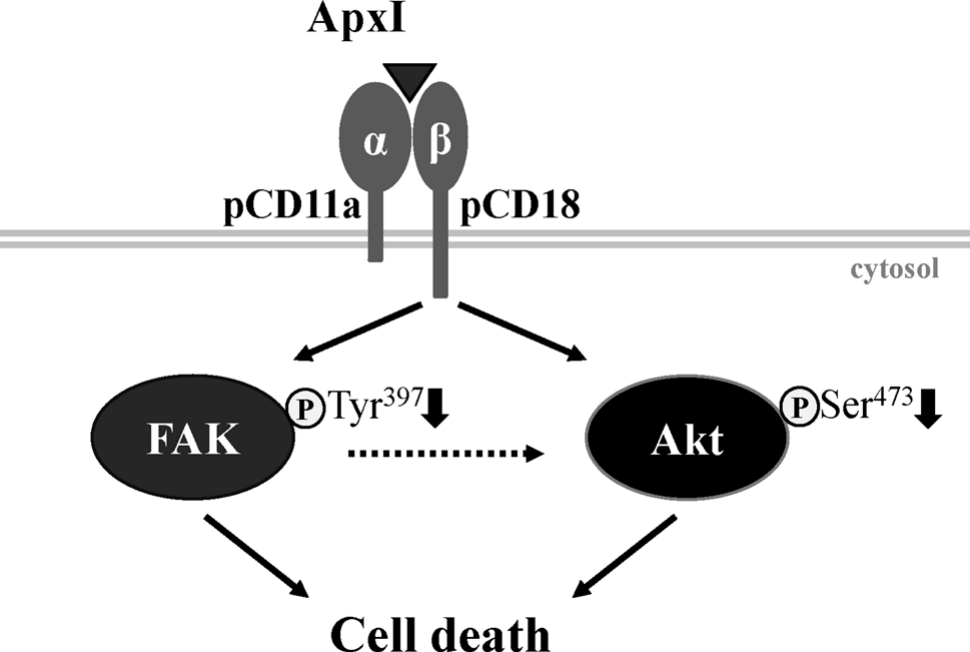
A schematic model illustrates the interaction of ApxI with LFA-1 and the signaling events affected by ApxI. Porcine LFA-1 (pCD11a/pCD18) serves as a potential receptor for ApxI. The CD18 subunit mediates the decreases in the levels of p-FAK^Tyr397^ and p-Akt^Sern^ in response to ApxI. Attenuation of FAK and Akt activity contributes to ApxI-induced cell death. Whether ApxI attenuates Akt activity in a FAK-dependent manner (dotted line) must be further investigated. The diagram was drawn using Microsoft PowerPoint 2013 (https://www.microsoft.com/).

In conclusion, the evidence presented in this study suggests that a signaling pathway consisting of the β_2_ integrin LFA-1, FAK, and Akt is essential for cell survival and attenuated by ApxI (Fig. 8). LFA-1 plays a pivotal role in ApxI-induced cell death, and FAK attenuation imparts this event. This study provides valuable insight into how ApxI impairs the host defense system via downregulation of β_2_ integrin-FAK survival signaling in porcine AMs.

## Supplementary Information

Supplementary Information.

## References

[1] Kamp EM, van Leengoed LA (1989). Serotype-related differences in production and type of heat-labile hemolysin and heat-labile cytotoxin of *Actinobacillus* (*Haemophilus*) *pleuropneumoniae*. J. Clin. Microbiol..

[2] Rosendal S (1988). Evaluation of heat-sensitive, neutrophil-toxic, and hemolytic activity of *Haemophilus* (*Actinobacillus*) *pleuropneumoniae*. Am. J. Vet. Res..

[3] Rycroft AN, Williams D, Cullen JM, Macdonald J (1991). The cytotoxin of *Actinobacillus pleuropneumoniae* (pleurotoxin) is distinct from the haemolysin and is associated with a 120 kDa polypeptide. J. Gen. Microbiol..

[4] Kuhnert P, Berthoud H, Straub R, Frey J (2003). Host cell specific activity of RTX toxins from haemolytic *Actinobacillus equuli* and *Actinobacillus suis*. Vet. Microbiol..

[5] Goldstein E, Lippert W, Warshauer D (1974). Pulmonary alveolar macrophage. Defender against bacterial infection of the lung. J. Clin. Invest..

[6] Chien MS (2009). *Actinobacillus pleuropneumoniae* serotype 10 derived ApxI induces apoptosis in porcine alveolar macrophages. Vet. Microbiol..

[7] Wu CM (2011). Mitogen-activated protein kinases p38 and JNK mediate *Actinobacillus pleuropneumoniae* exotoxin ApxI-induced apoptosis in porcine alveolar macrophages. Vet. Microbiol..

[8] Chang NY (2014). Elucidating the role of ApxI in hemolysis and cellular damage by using a novel *apxIA* mutant of *Actinobacillus pleuropneumoniae* serotype 10. J. Vet. Sci..

[9] Ambagala TC, Ambagala AP, Srikumaran S (1999). The leukotoxin of *Pasteurella haemolytica* binds to β_2_ integrins on bovine leukocytes. FEMS Microbiol. Lett..

[10] Kieba IR (2007). *Aggregatibacter actinomycetemcomitans* leukotoxin requires β-sheets 1 and 2 of the human CD11a β-propeller for cytotoxicity. Cell. Microbiol..

[11] Lally ET (1997). RTX toxins recognize a β_2_ integrin on the surface of human target cells. J. Biol. Chem..

[12] Morova J, Osicka R, Masin J, Sebo P (2008). RTX cytotoxins recognize β_2_ integrin receptors through N-linked oligosaccharides. Proc. Natl. Acad. Sci. USA.

[13] Guermonprez P (2001). The adenylate cyclase toxin of *Bordetella pertussis* binds to target cells via the α_M_β_2_ integrin (CD11b/CD18). J. Exp. Med..

[14] Jeyaseelan S (2000). Lymphocyte function-associated antigen 1 is a receptor for *Pasteurella haemolytica* leukotoxin in bovine leukocytes. Infect. Immun..

[15] Vanden Bergh PG, Zecchinon LL, Fett T, Desmecht D (2009). Porcine CD18 mediates *Actinobacillus pleuropneumoniae* ApxIII species-specific toxicity. Vet. Res..

[16] Chen ZW (2011). Mechanisms underlying *Actinobacillus pleuropneumoniae* exotoxin ApxI induced expression of IL-1β, IL-8 and TNF-α in porcine alveolar macrophages. Vet. Res..

[17] Hsu CW (2016). Involvement of NF-κB in regulation of *Actinobacillus pleuropneumoniae* exotoxin ApxI-induced proinflammatory cytokine production in porcine alveolar macrophages. Vet. Microbiol..

[18] Arnaout MA (2016). Biology and structure of leukocyte β_2_ integrins and their role in inflammation. F1000Res.

[19] Fan Z, Ley K (2015). Leukocyte arrest: biomechanics and molecular mechanisms of β_2_ integrin activation. Biorheology.

[20] Vachon PH (2011). Integrin signaling, cell survival, and anoikis: distinctions, differences, and differentiation. J. Signal Transduct..

[21] Spencer JP, Rice-Evans C, Williams RJ (2003). Modulation of pro-survival Akt/protein kinase B and ERK1/2 signaling cascades by quercetin and its in vivo metabolites underlie their action on neuronal viability. J. Biol. Chem..

[22] Ahmad JN (2016). cAMP signalling of *Bordetella* adenylate cyclase toxin through the SHP-1 phosphatase activates the BimEL-Bax pro-apoptotic cascade in phagocytes. Cell. Microbiol..

[23] Wiles TJ, Dhakal BK, Eto DS, Mulvey MA (2008). Inactivation of host Akt/protein kinase B signaling by bacterial pore-forming toxins. Mol. Biol. Cell.

[24] Atapattu DN, Czuprynski CJ (2005). *Mannheimia haemolytica* leukotoxin induces apoptosis of bovine lymphoblastoid cells (BL-3) via a caspase-9-dependent mitochondrial pathway. Infect. Immun..

[25] Hynes RO (2002). Integrins: bidirectional, allosteric signaling machines. Cell.

[26] El Kebir D, Filep JG (2013). Modulation of neutrophil apoptosis and the resolution of inflammation through β_2_ integrins. Front. Immunol..

[27] Oellerich T (2013). Front β_2_ integrin-derived signals induce cell survival and proliferation of AML blasts by activating a Syk/STAT signaling axis. Blood.

[28] Weber KS, York MR, Springer TA, Klickstein LB (1997). Characterization of lymphocyte function-associated antigen 1 (LFA-1)-deficient T cell lines: the α_L_ and β_2_ subunits are interdependent for cell surface expression. J. Immunol..

[29] Parsons JT (2003). Focal adhesion kinase: the first ten years. J. Cell Sci..

[30] Park SK (2009). Kalopanaxsaponin A inhibits PMA-induced invasion by reducing matrix metalloproteinase-9 via PI3K/Akt- and PKCδ-mediated signaling in MCF-7 human breast cancer cells. Carcinogenesis.

[31] Kawakami Y (2004). Protein kinase C βII regulates Akt phosphorylation on Ser-473 in a cell type- and stimulus-specific fashion. J. Biol. Chem..

[32] Hsu AH (2018). Crosstalk between PKCα and PI3K/AKT signaling is tumor suppressive in the endometrium. Cell Rep..

[33] Li L, Sampat K, Hu N, Zakari J, Yuspa SH (2006). Protein kinase C negatively regulates Akt activity and modifies UVC-induced apoptosis in mouse keratinocytes. J. Biol. Chem..

