# Supplementary material for: Actinobacillus pleuropneumoniae exotoxin ApxI induces cell death via attenuation of FAK through LFA-1
Source: Sci Rep. 2021 Jan 18;11:1753. doi: 10.1038/s41598-021-81290-9 (PMC7813829; doi:10.1038/s41598-021-81290-9)
Supplement: Supplementary file 1 — Supplementary Information. [file 41598_2021_81290_MOESM1_ESM.pdf]

Supplementary information

***Actinobacillus pleuropneumoniae* exotoxin ApxI induces cell death via attenuation of FAK through LFA-1**

Siou-Cen Li[1,2], Yu-Tsen Cheng[1], Ching-Yang Wang[1], Jia-Ying Wu[1], Zeng-Weng Chen[2], Jyh-Perng Wang[2], Jiunn-Horng Lin[2], and Shih-Ling Hsuan[1,*]

[1]Graduate Institute of Veterinary Pathobiology, College of Veterinary Medicine, National Chung Hsing University, Taichung City 402, Taiwan
[2]Animal Technology Laboratories, Agricultural Technology Research Institute, Hsinchu City 300, Taiwan
*Corresponding author

Supplementary Figures

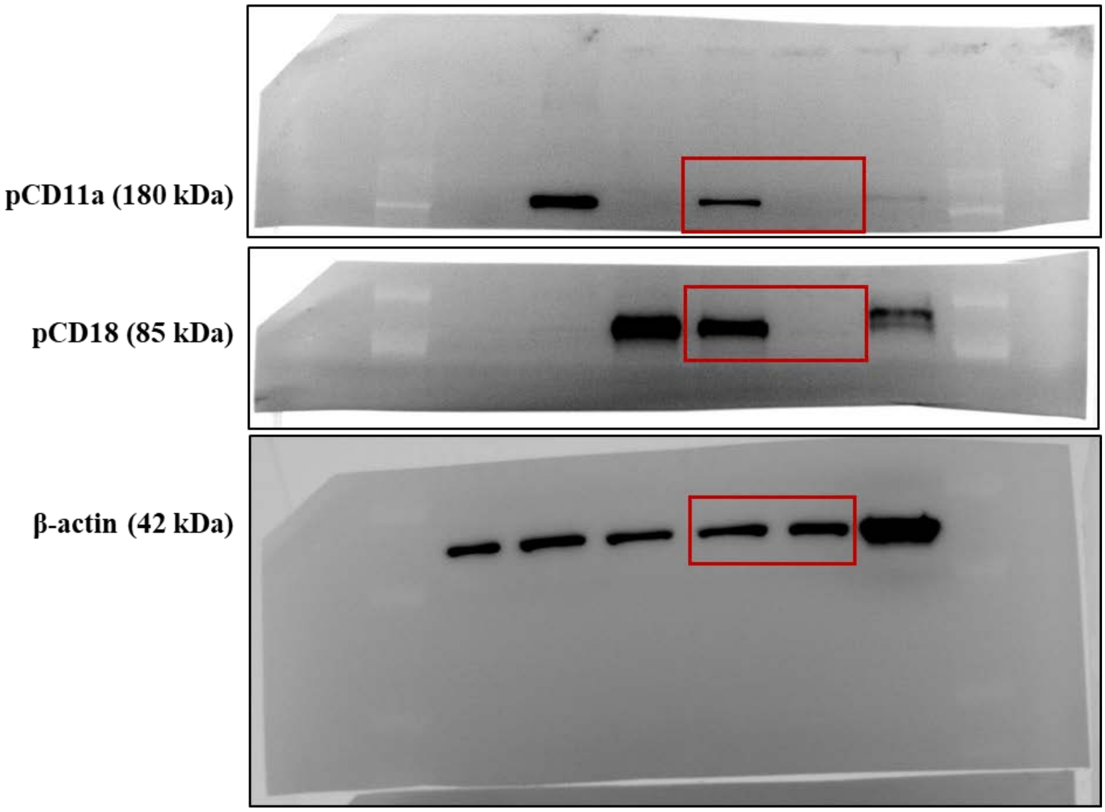

Supplementary Figure S1. The figure shows the original uncropped Western blot images for Fig. 1c.

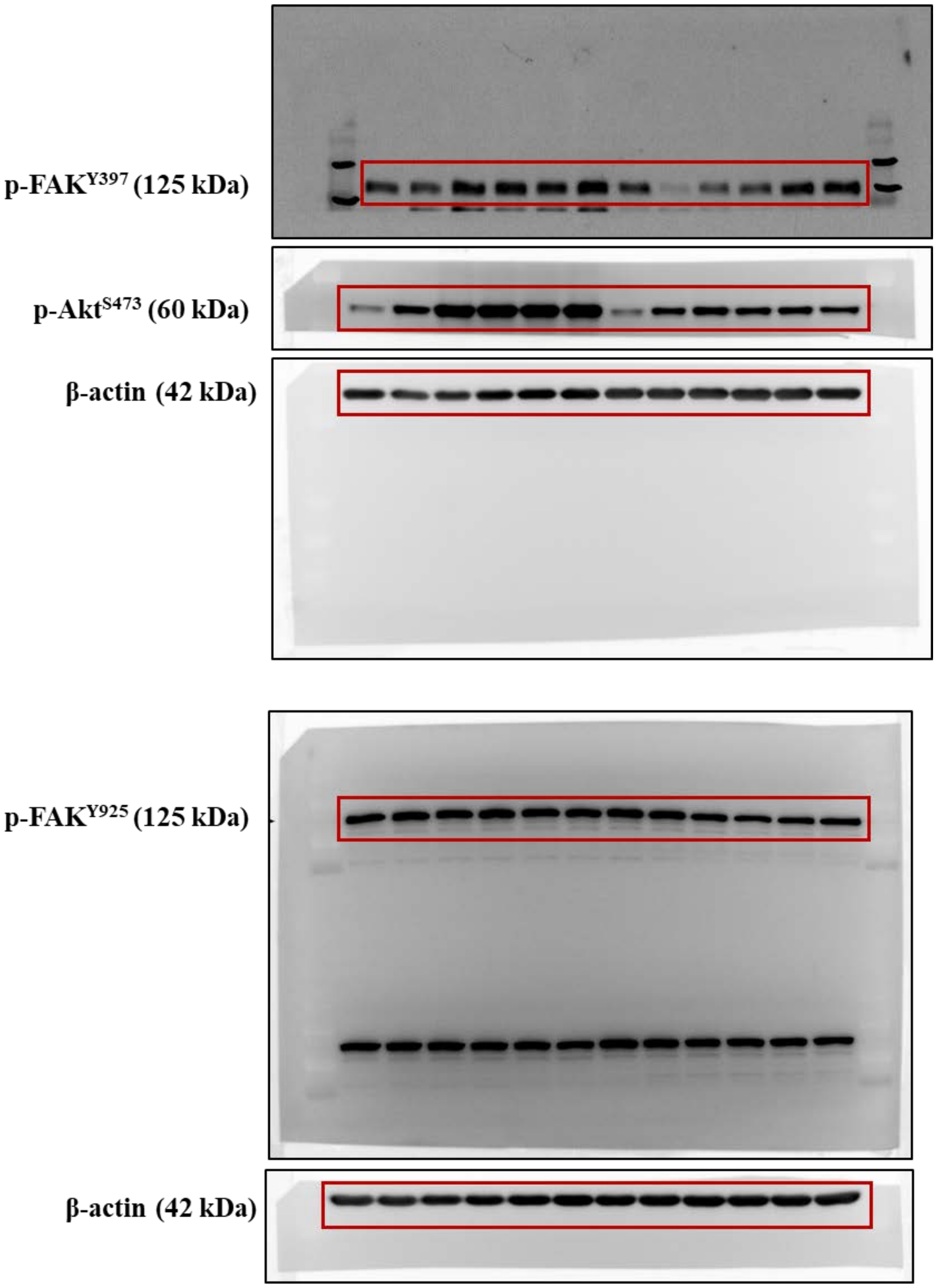

Supplementary Figure S2. The figure shows the original uncropped Western blot images for Fig. 3a. The blot of p-FAK^{Y925} showed specific bands from two experiments, that full-size gels were cut horizontally into gel strips prior to transfer to the membrane in order to detect the same target protein in samples.

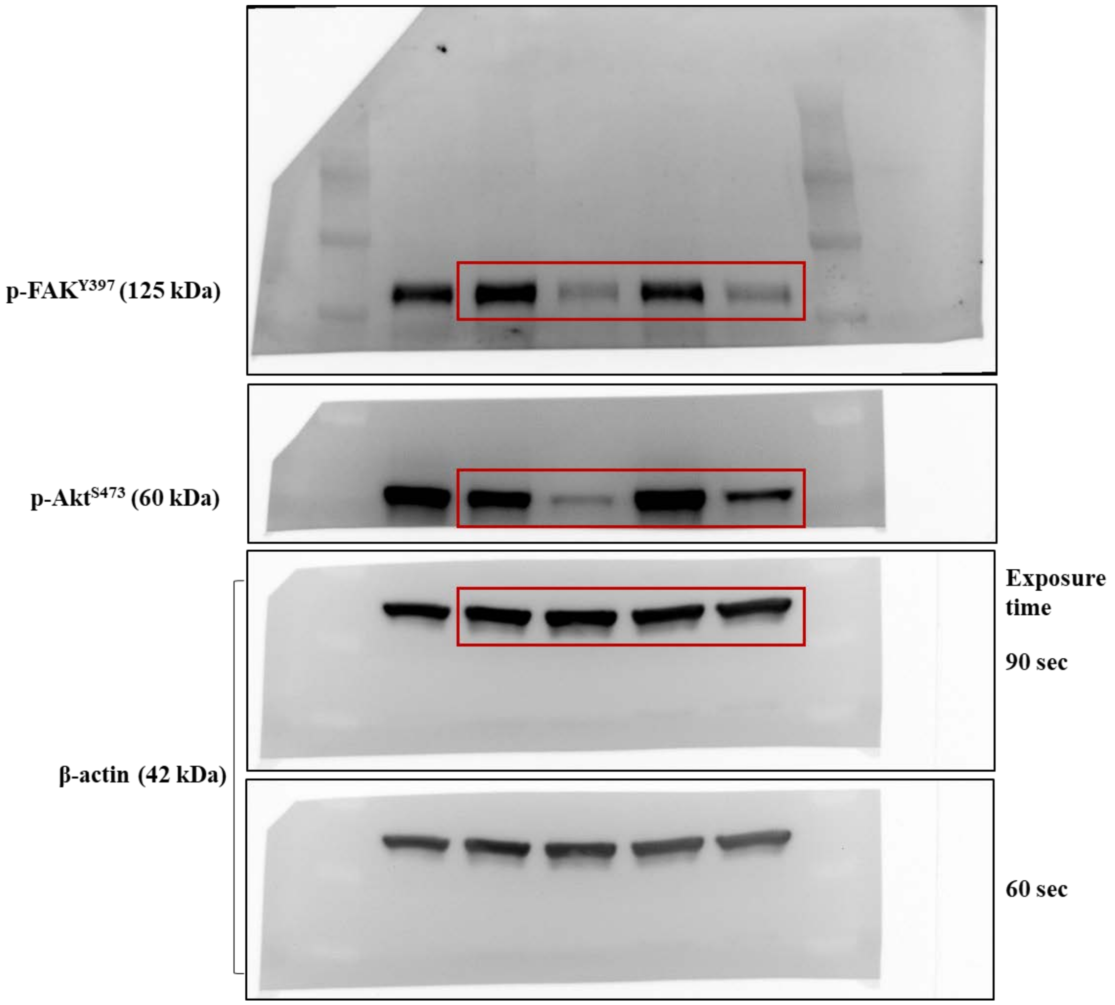

Supplementary Figure S3. The figure shows the original uncropped Western blot images for Fig. 4a.

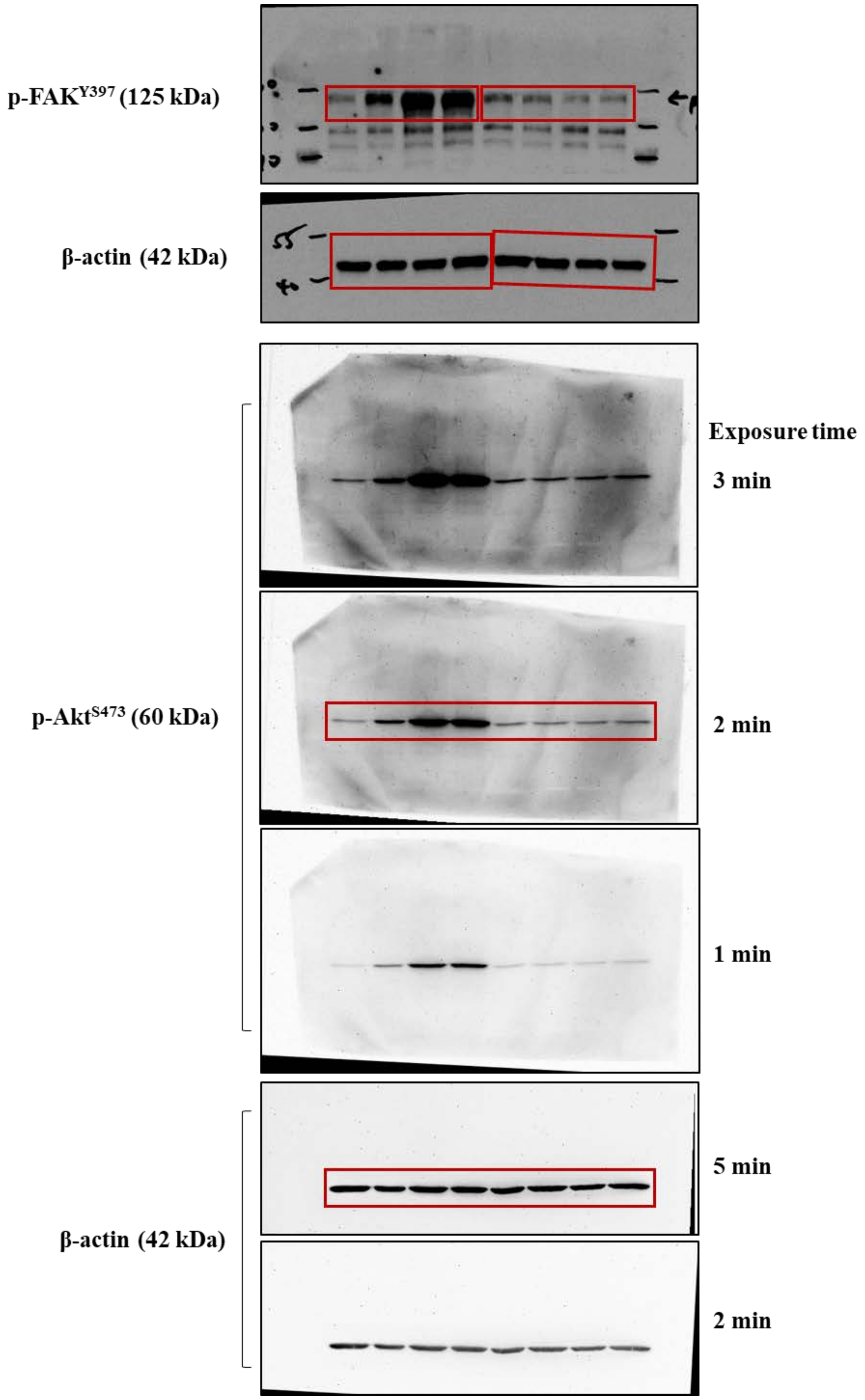

Supplementary Figure S4. The figure shows the original uncropped Western blot images for Fig. 5a.

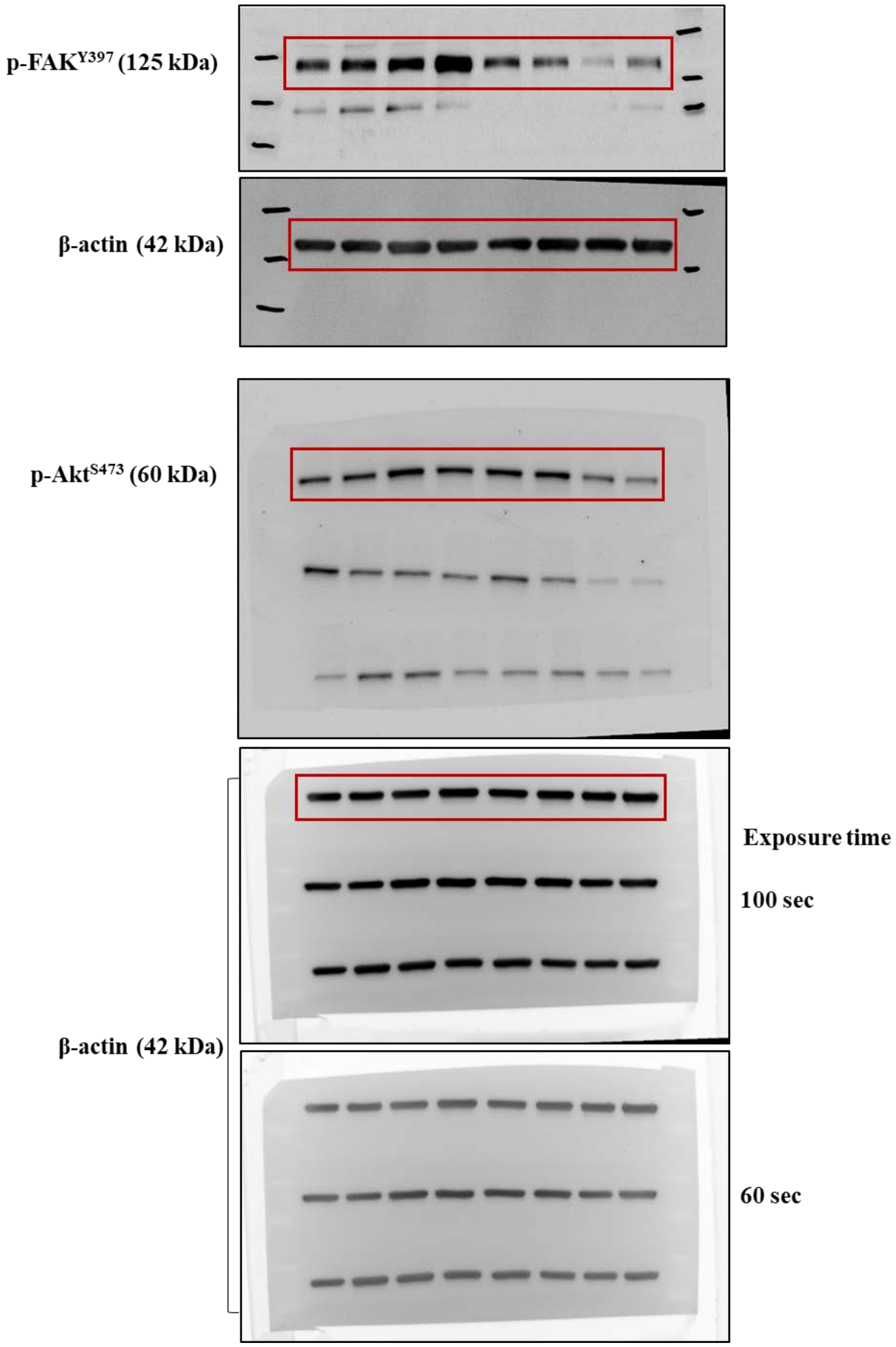

Supplementary Figure S5. The figure shows the original uncropped Western blot images for Fig. 5d. The blots of p-Akt^{S473} and β-actin showed specific bands from three experiments, that full-size gels were cut horizontally into gel strips prior to transfer to the membranes in order to detect the same target protein in samples.

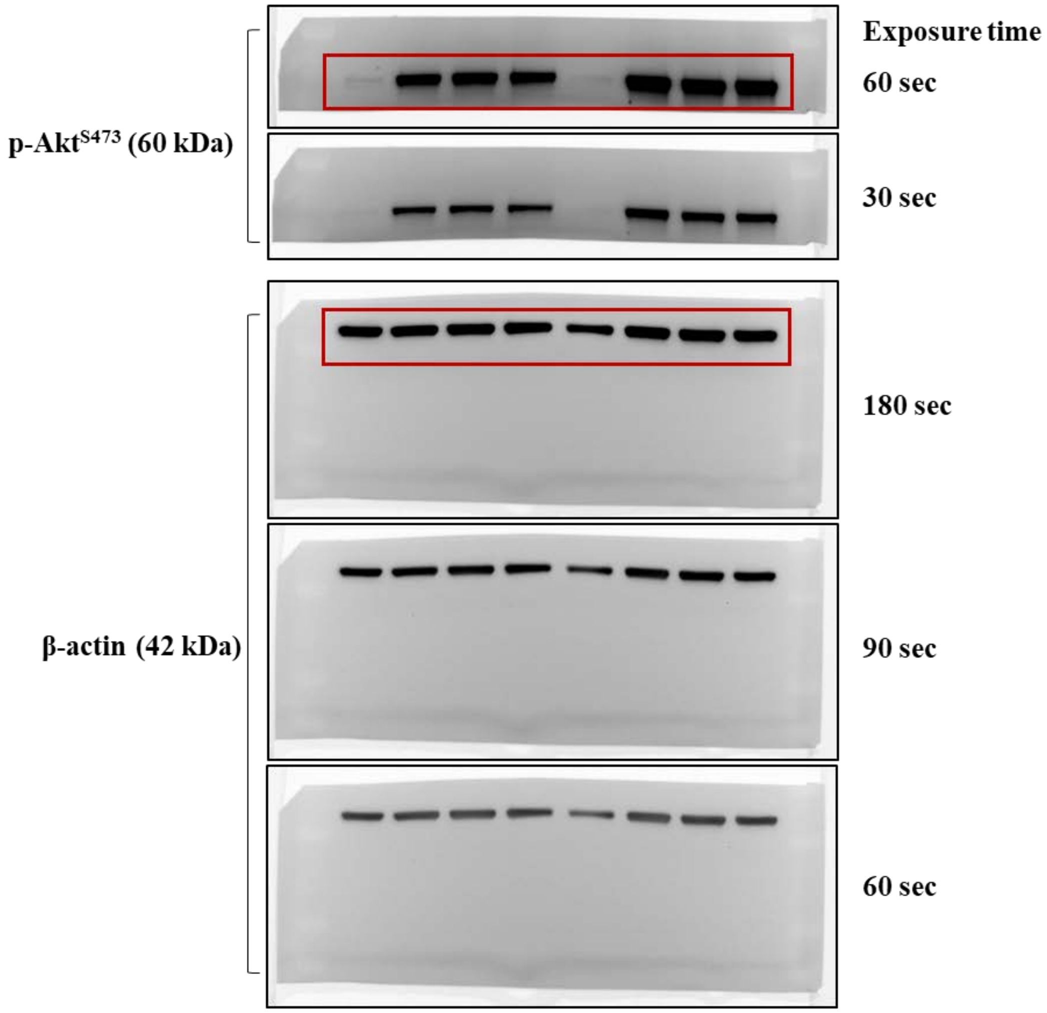

Supplementary Figure S6. The figure shows the original uncropped Western blot images for Fig. 6a.

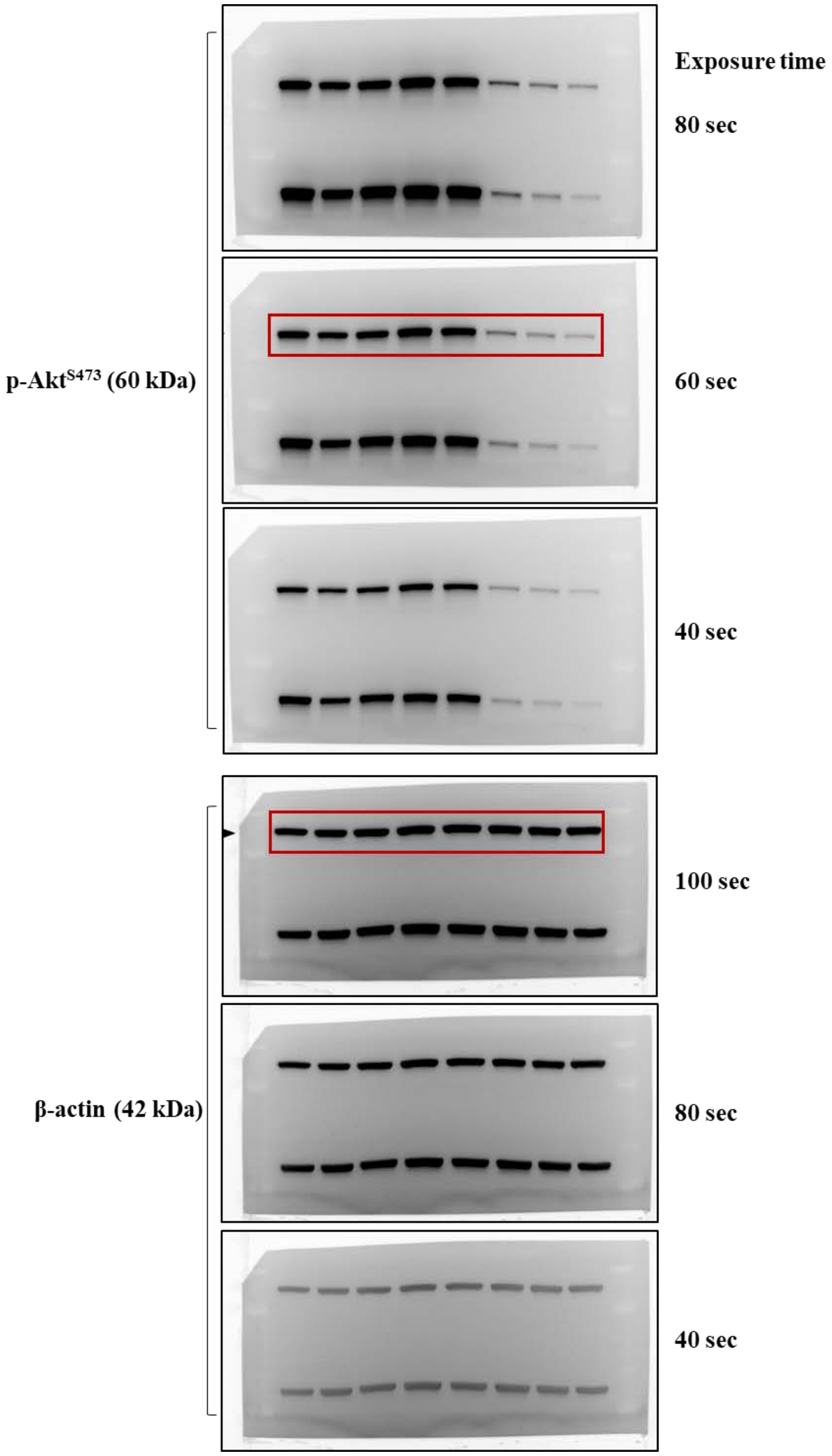

Supplementary Figure S7. The figure shows the original uncropped Western blot images for Fig. 6c. The blots of p-Akt^{S473} and β-actin showed specific bands from two experiments, that full-size gels were cut horizontally into gel strips prior to transfer to the membranes in order to detect the same target protein in samples.

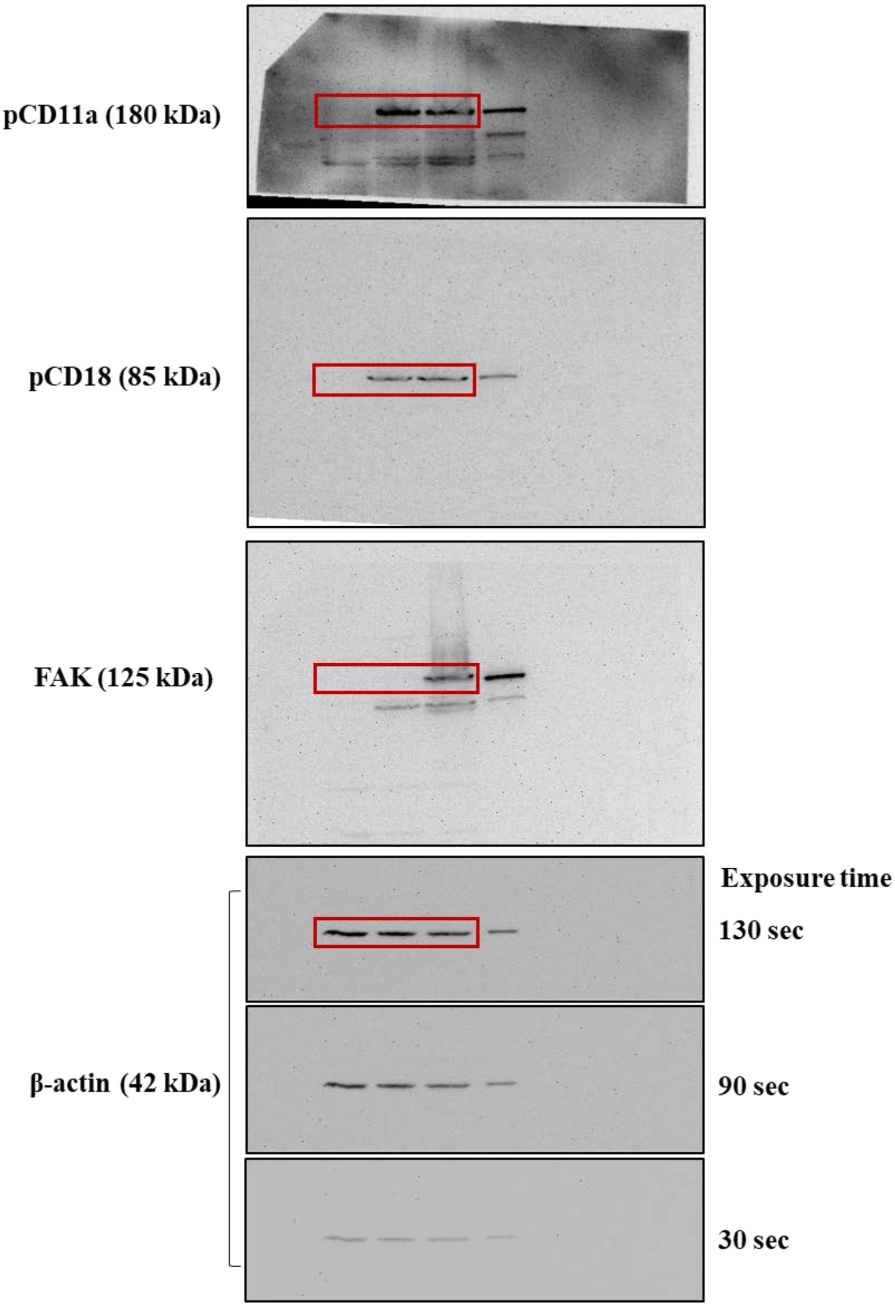

Supplementary Figure S8. The figure shows the original uncropped Western blot images for Fig. 7b.
